# Maltodextrin from Sweet Cassava: A Promising Endurance Enhancer

**DOI:** 10.3390/foods13050766

**Published:** 2024-03-01

**Authors:** Kakanang Posridee, Anant Oonsivilai, Ratchadaporn Oonsivilai

**Affiliations:** 1 School of Food Technology, Institute of Agricultural Technology, Suranaree University of Technology, Nakhon Ratchasima 30000, Thailand; posridee.ka@gmail.com; 2 School of Electrical Engineering, Institute of Engineering, Suranaree University of Technology, Nakhon Ratchasima 30000, Thailand; 3 Health and Wellness Research Unit, Suranaree University of Technology, Nakhon Ratchasima 30000, Thailand

**Keywords:** sweet cassava, maltodextrin, endurance, Wistar rat

## Abstract

The effects of maltodextrin and crude extract from sweet cassava on exercise endurance were examined in the male Wistar rat. The rats were randomly assigned to either an exercise training group or a non-exercise training group. Both groups were further divided into subgroups that received either a control, crude extract (250 or 500 mg/kg), or maltodextrin (250 or 500 mg/kg) orally once daily for 16 days. The time to the point of exhaustion after weight-loaded forced swimming was measured on day 16. Body weight gain, relative organ weight, biochemical parameters, and liver and gastrocnemius muscle glycogen content were also determined. Maltodextrin at a dose of 500 mg/kg significantly increased the time to the point of exhaustion compared to all other groups. Maltodextrin and crude extract with both doses significantly increased liver and gastrocnemius muscle glycogen content compared to the control group. There were no significant differences in glucose, BUN, triglyceride, or insulin levels between the groups. Crude extract at a dose of 250 mg/kg significantly increased AST and ALT levels, and LDH levels significantly increased in the exercise training group. Creatinine levels were significantly higher in the exercise training group compared to the non-exercise training group. Exercise boosted antioxidant enzymes, glycogen, and reduced damaging free radicals in the rats. Maltodextrin and crude extract further amplified this effect by activating AMPK and PGC-1α, suggesting that they combat fatigue through an antioxidant pathway linked to AMPK. These findings suggest that maltodextrin and crude extract from sweet cassava may have the potential to enhance exercise endurance. They may increase glycogen storage in the liver and gastrocnemius muscle, potentially through improved glycogen reserves and glycogen sparing effects. Further studies are needed to elucidate the mechanisms underlying these effects.

## 1. Introduction

Sweet cassava (*Manihot esculenta* Crantz.) is a starchy root crop that is a staple food for millions of people in tropical and subtropical regions around the world. It is a good source of carbohydrates, vitamins, and minerals. Sweet cassava is also a good source of resistant starch, a type of carbohydrate that is not digested in the small intestine and can have beneficial effects on gut health [1,2]. Furthermore, research has shown that dietary fiber from cassava pulp offers health advantages, including cholesterol and bile acid binding, and prebiotic activity [3]. Endurance exercise is any type of physical activity that requires sustained effort over a period of time. Endurance athletes, such as runners, cyclists, and swimmers, need to have high levels of cardiovascular fitness and muscular endurance. Several studies have investigated the effects of sweet cassava on endurance performance. These studies have shown that sweet cassava can help to improve endurance in both rats and humans.

Ogawa et al. [4] found that rats supplemented with sweet cassava polysaccharides (SCP) were able to run for a significantly longer time before exhaustion than rats that were not supplemented with SCP. The SCP-supplemented rats also had higher levels of muscle glycogen, which is a stored form of energy that is used during exercise. In addition, Fukuda et al. [5] found that rats supplemented with SCP had lower levels of blood lactate after exercise than rats that were not supplemented with SCP. Blood lactate is a byproduct of exercise that can contribute to fatigue. With regard to studies in humans, Nishii et al. [6] found that human runners who consumed a meal containing SCP before running were able to run for a significantly longer time than runners who consumed a meal without SCP. Furthermore, Costa et al. [7] found that human cyclists who consumed a drink containing SCP during a cycling race had lower levels of perceived exertion and higher levels of power output than cyclists who consumed a drink without SCP.

Overall, the available evidence suggests that sweet cassava can be a beneficial supplement for endurance athletes. SCP can help to improve endurance performance by increasing muscle glycogen stores, reducing blood lactate levels, and reducing perceived exertion. However, more research is needed to confirm these findings and to determine the optimal dosage of SCP for endurance in athletes. It is important to note that sweet cassava can contain cyanogenic glycosides, which are toxic compounds that can cause cyanide poisoning if consumed in large amounts. Therefore, it should be consumed only when properly prepared.

In addition to its potential benefits for endurance exercise, sweet cassava also has a number of other health benefits. It is a good source of fiber, which can help to promote digestive health. Sweet cassava is also a good source of antioxidants, which can help to protect cells from damage.

While research suggests maltodextrin may be promising in regard to enhancing endurance performance in some athletes [8], the picture is not definitive. Studies show mixed results, with some finding no significant benefit compared to other carbs or a placebo [9]. Timing and dosage seem crucial [10], and individual needs and exercise type play a role.

Carbohydrate oral rinsing with maltodextrin shows some early evidence for performance improvement [11] but requires further investigation. Additionally, while maltodextrin may help maintain muscle glycogen during extended exercise [12], more research is needed to confirm this benefit.

Maltodextrin should not be considered a panacea. It is safe but it can cause gastrointestinal distress in some cases [13]. Carbohydrate sources like fruits and vegetables should also be considered for their additional nutrients. Most importantly, proper training and a balanced diet remain the basis for peak athletic performance [14].

This study aimed to investigate the impact of sweet cassava on athletic performance, specifically comparing it to maltodextrin, which is another carbohydrate source. Using rats as a model, the researchers examined how both maltodextrin and crude extract of sweet cassava affected the animals’ exercise time to the point of exhaustion, as well as their blood glucose and insulin levels.

## 2. Materials and Methods

### 2.1. Maltodextrin and Crude Extract Solution Preparation

Sweet cassava flour (250 g) was mixed with 500 mL of water and stirred overnight. The mixture was centrifuged at 14,300× *g*, at 4 °C for 20 min, and the supernatant was collected. The extraction was then repeated using the sediment and a second supernatant was obtained. The two supernatants were combined freeze-dried. The powder was kept in a vacuum container at −20 °C until used.

Maltodextrin was used as a reference for comparison and prepared in the same way. Maltodextrin and crude extract from sweet cassava were used throughout the experiments described in this study. Fresh solutions of these materials were prepared on the day of each experiment by dissolving them in DDD water at the required concentrations.

### 2.2. Chemicals

A 95% ethanol solution (made with Analytical Grade ethanol of at least 99.8% purity (CarloErba Reagents, Val-de-Reuil, France) was used. A 5% phenol solution was created by mixing 50 g of phenol (Sigma Chemical Co., St. Louis, MO, USA) with solvent. A 30% potassium hydroxide and sodium sulfate solution was prepared by adding 180 g of potassium hydroxide (Sigma Chemical Co., St. Louis, MO, USA) as a solvent. A stock glycogen standard solution was made by dissolving 50 milligrams of cattle glycogen powder (Sigma Co., St. Louis, MO, USA) in 10 milliliters of solvent to reach a concentration of 5 milligrams per milliliter.

### 2.3. Experimental Designs

Eight-week-old male Wistar rats weighing 250–300 g were housed in a controlled laboratory environment. This environment included a 12 h light/dark cycle, a consistent temperature of 25 ± 1 °C, and unrestricted access to food and water. All experiments adhered to the animal care and use committee guidelines of Suranaree University of Technology (SUT) and were conducted under a permit issued by the SUT Animal Care and Use Committee (Approved Code 21/2014, approved date: 2 October 2014).

Following a seven-day period for the rats to adjust to their new environment, the researchers randomly divided 50 rats into five distinct groups. The first group, the control group, received solely 1 mL/kg of distilled, deionized water (DDD water) each day. The remaining four groups of rats were designated as treatment groups and received different dosages of maltodextrin and crude extract administered directly into their stomachs using a feeding tube, known as gavage. The low-dose group received 250 mg/mL/kg of the mixture, while the high-dose group received 500 mg/mL/kg. These specific dosages were chosen based on previous research conducted by Yen et al. [15], which calculated the conversion of a human dose used in traditional methods to a rodent dose based on the following criteria:

Rat dose (mg/kg) = (human dose mg/kg × 70 kg × 0.018)/2002.2

#### 2.3.1. Exercise Endurance Capacity

The non-exercise groups of rats were kept in cages with 3 cm of water to exclude potential stress and other deleterious effects. All the rats were allowed to swim until exhaustion on the 16th day after receiving their respective treatments. Their swimming time was then recorded.

In this study, rats underwent a 15-day training period. Those in the exercise groups received vehicles, maltodextrin, and crude extract at doses of 250 and 500 mg/mL. Their training protocol consisted of 30 min of swimming per day, gradually increasing in duration until it reached 1 h. The swimming took place in an acrylic plastic pool measuring 90 cm × 45 cm × 45 cm, filled with water up to 60 cm and maintained at a temperature between 34 and 36 °C. In the meantime, the non-exercise groups received the same dosages of vehicles, crude extract, and maltodextrin but were kept in cages with only 3 cm of water to avoid potential stress or deleterious effects [16].

#### 2.3.2. Determination of Blood Biochemical Variables

When the rats were exhausted from their ordeal, they were euthanized with carbon dioxide. The researchers swiftly collected 10 mL of blood samples from their hearts and dissected specific hind-limb muscles (soleus, EDL, and gastrocnemius), and liver tissue. After drying with filter paper and careful weighing, these tissues were flash-frozen on dry ice at −20 °C to preserve their state for future biochemical or histological analysis.

After collecting whole blood via cardiac puncture, the researchers separated the serum and plasma. Serum samples, devoid of anticoagulant, were cooled for 3.5 h at 4 °C, then centrifuged to extract the serum for lactate dehydrogenase (LDH) analysis. Plasma samples, containing anticoagulant, underwent faster centrifugation (Labconco Corporation Ltd., Kansas City, MO, USA) and were analyzed for aminotransferase (ALT), glucose, creatinine, and lactate dehydrogenase (LDH), which were determined using an automated analyzer [17].

#### 2.3.3. Determination of Glycogen Levels in Tissue Samples

This study employed a modified version of the previously described method by Lo et al. [18] for measuring glycogen levels in liver and gastrocnemius muscle samples. Briefly, tissue samples weighing 25 mg were weighed and placed in Eppendorf tubes on ice. To ensure complete immersion, 750 μL of 30% KOH saturated with Na_2_SO_4_ was added to each tube. The samples were then heated in a boiling water bath for 30 min until a homogeneous solution was obtained. Following cooling, 900 μL of 95% ethanol was added to each tube to precipitate glycogen from the alkaline digestate. After standing on ice for 30 min, the samples were centrifuged at 840× *g* for 30 min. The supernatant was then removed, and the tubes were dried upside down for 5 min. The glycogen precipitate was then dissolved in 1.5 mL of double-distilled water. Aliquots of the solution (0.5 mL each) were dispensed into three test tubes, followed by 0.5 mL of 5% phenol solution. Subsequently, 2.5 mL of concentrated sulfuric acid (96–98%) was rapidly added to each tube while ensuring proper mixing. After 10 min, the tubes were shaken and placed in a water bath at 25–30 °C for 10–20 min before absorbance was measured at 490 nm on a spectrophotometer. Standard glycogen solutions (0–200 μg/mL) and a blank were prepared for comparison. All tests were performed in triplicate to minimize errors.

The equation used to calculate the tissue content of glycogen was as follows:

$$grams\;of\;\frac{glycogen}{100}g\;tissue=\frac{A490}{k}\times \frac{V}{v}\times \frac{10^{-4}}{W}$$

where the following abbreviations are used:

V = total volume of glycogen solution
v = volume of aliquot used in the color reaction
A490 = absorbance at 490 nm
W = weight of tissue sample in grams
k = slope of standard curve
Units = 1 per microgram glycogen.

#### 2.3.4. Oxidative Stress-Related Parameter Analysis

As soon as the rats reached the point of exhaustion, they were put under anesthesia with carbon dioxide. The researchers then swiftly dissected specific muscles (soleus, EDL, and gastrocnemius) from both the legs and the liver of each rat. These tissues were carefully dried, weighed, and then flash-frozen on dry ice to preserve their state for future analysis. Meanwhile, the liver and muscle of a separately sacrificed rat were dissected, washed thoroughly with ice-cold saline solution, and prepared as homogenized samples in double-distilled water. These prepared samples were used to measure the levels of reactive oxygen species (ROS), malondialdehyde (MDA), and superoxide dismutase (SOD) in the liver.

#### 2.3.5. Reactive Oxygen Species (ROS)

The amount of hydrogen peroxide (H_2_O_2_) produced was determined using the Holland and Storey’s method [19]. Briefly, a 0.1 mL liver extract was added to a reaction mixture containing specific concentrations of potassium chloride, potassium phosphate, magnesium chloride, EDTA, Tris-HCl, and acetylated ferrocytochrome c. The oxidation of ferrocytochrome c, which serves as an indicator of H_2_O_2_ production, was monitored at 550 nm using a spectrophotometer.

#### 2.3.6. Malondialdehyde (MDA)

Oxidative stress levels were assessed through lipid peroxidation (LPO) using the method of Torun et al. [20]. Malondialdehyde (MDA), a byproduct of lipid peroxidation, was used as a marker of oxidative stress intensity. The reaction of MDA with thiobarbituric acid formed a colored product which was measured at 532 nm by a spectrophotomer. LPO levels were reported as millimoles of MDA per milligram of protein.

#### 2.3.7. Superoxide Dismutase (SOD)

The amount of chromic acetate generated served as a measure of superoxide dismutase (SOD) activity in this assay. SOD activity was determined colorimetrically at 450 nm using the method of Marklund [21].

#### 2.3.8. RNA Isolation and Reverse Transcription (RT)-PCR

Total RNA was isolated from liver and muscle samples using a NucleoSpin RNA kit (Macherey-Nagel, Hoerdt, France) following the manufacturer’s protocol. The isolated RNA was reverse transcribed into cDNA using qPCR RT Master mix (Vivantis Technologies, Shah Alam, Malaysia) as per the manufacturer’s instructions. Gene expression analysis was performed using qPCRBIO SyGreen Mix Lo-ROX (PCR Biosystems, London, UK) according to the manufacturer’s recommendations on a Biorad/C1000Touch Thermocycler (Hercules, CA, USA) with specific primers targeting AMPK α1, AMPK α2, PGC-1α, and GAPDH sequences (Table S1). GAPDH was used as the reference gene for relative mRNA quantification. Real-time PCR was conducted using DNA.

Engine Optical 2 was used with distinct thermal cycling parameters for each target gene: AMPK α1/α2 (50 °C/30 min, 95 °C/10 min, 40 cycles of 95 °C/30 s, 56 °C/1 min, 72 °C/50 s), and PGC-1α/GAPDH (50 °C/30 min, 95 °C/10 min, 40 cycles of 95 °C/30 s, 60 °C/1 min, 72 °C/50 s).

### 2.4. Statistical Analysis

The data are presented as mean values ± standard error of the mean (SEM). Statistical analysis was conducted using analysis of variance (ANOVA) in SPSS version 18.0 (SPSS Inc., Chicago, IL, USA). Post hoc comparisons between groups were performed with Duncan’s test. A *p*-value less than 0.05 (*p* < 0.05) was considered statistically significant. All graphs were generated using SigmaPlot software (version 10, Systat Software Inc., Richmond, CA, USA).

## 3. Results

### 3.1. Effects of Maltodextrin (M) and Crude Extract (CP) from Sweet Cassava on Physiology

#### 3.1.1. Exercise Endurance

**Figure 1:**
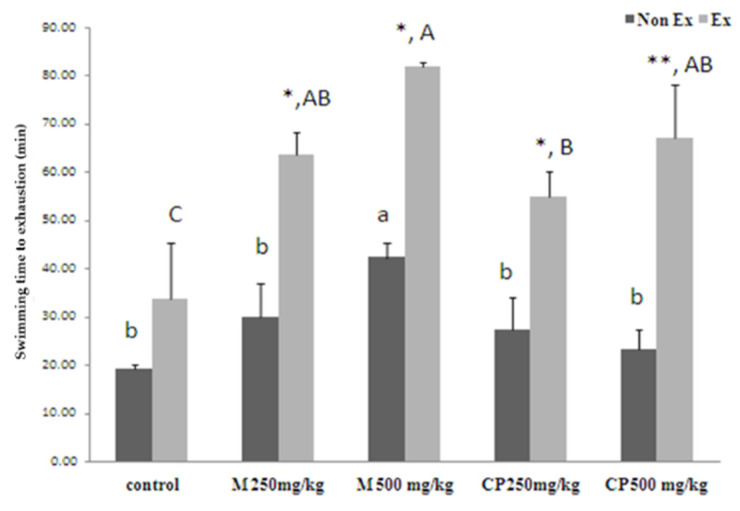
Effects of 16 days of administration of maltodextrin (M) and crude extract (CP) from sweet cassava on exercise endurance. Values are expressed as mean ± SEM; *n* = 5 per group. * indicates a significant difference between Non Ex and Ex groups with the same treatment (*p* < 0.05, two-way ANOVA; Duncan’s method). ** indicates significant difference between Non Ex and Ex groups with the same treatment (*p* < 0.01, two-way ANOVA; Duncan’s method). Small letters indicate significant differences within Non Ex groups. Capital letters indicate significant differences within Ex groups. Means sharing the same superscript are not significantly different from each other.

This study investigated the impact of sweet cassava extract and maltodextrin on exercise endurance in male Wistar rats, with and without exercise training. The rats were administered maltodextrin and crude extract at doses of 250 mg/kg and 500 mg/kg for 16 days, followed by an exercise test to the point of exhaustion. The results are shown in Figure 1. In the non-exercise training group, the rats that received maltodextrin at a dose of 500 mg/kg exhibited significantly longer swimming times to the point of exhaustion (42 min) compared to all the other groups (*p* < 0.05). In the exercise training group, the rats that received maltodextrin at a dose of 500 mg/kg displayed significantly longer swimming times to the point of exhaustion (82 min) compared to the crude extract 500 (67 min), maltodextrin 250 mg/kg (63 min), and maltodextrin 250 mg/kg (54 min) treatments, respectively (*p* < 0.05).

#### 3.1.2. Body Weight Gain

**Figure 2:**
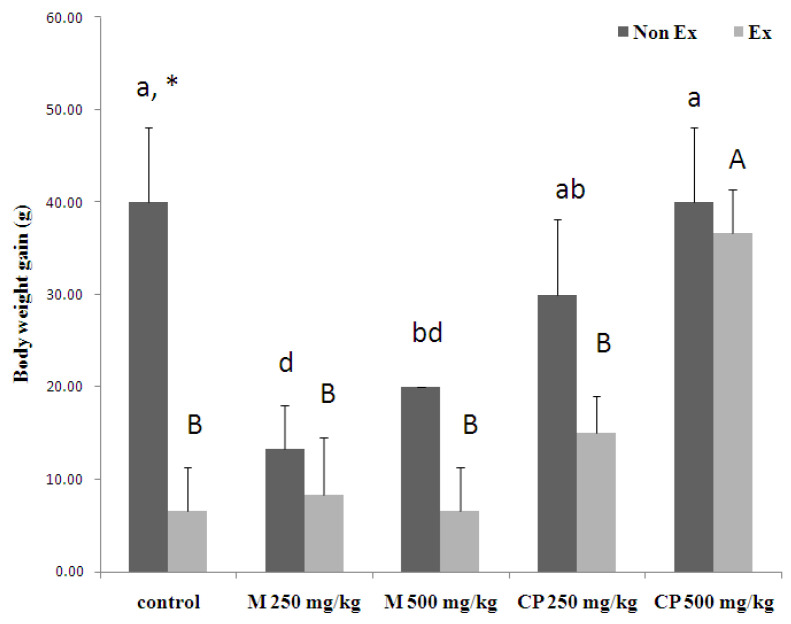
The effects of sixteen days of maltodextrin (M) and crude extract (CP) from sweet cassava on the body weight gain of the rats. Values are shown as mean ± SEM, with five rats per group. An asterisk (*) indicates a significant difference between non-exercise (Non Ex) and exercise (Ex) groups receiving the same treatment. Small letters denote significant differences within the non-exercise group, while capital letters represent significant differences within the exercised group. Means sharing the same superscript are not statistically different from each other (*p* < 0.05, two-way ANOVA; Duncan’s method).

The changes in body weight of the male Wistar rats from day 1 to day 16 after receiving crude extract and maltodextrin with and without exercise training are shown in Figure 2.

In the non-exercise training group, the rats receiving maltodextrin and crude extract showed a decrease in weight gain compared to the control group. However, in the exercise training group, the weight gain of rats administered crude extract at a dose of 500 mg/kg was significantly higher than for all the other groups (*p* < 0.05). Furthermore, a comparison of exercise and non-exercise training within the control group revealed a significant difference in body weight gain.

**Figure 3:**
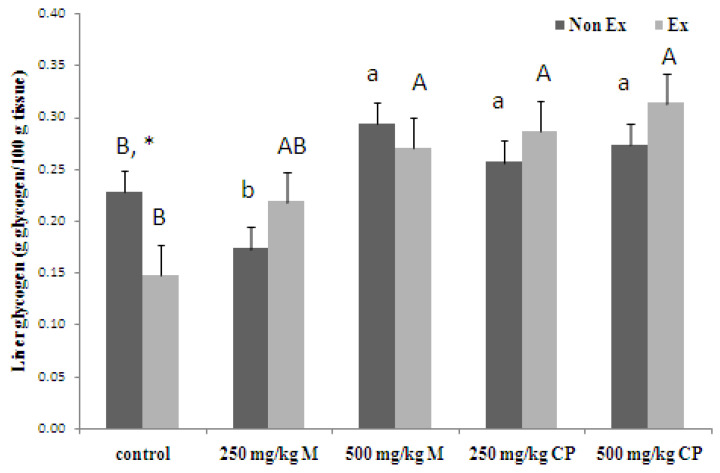
Effects of 16 days administration of maltodextrin (M) and crude extract (CP) from sweet cassava on liver glycogen content. Values are expressed as mean ± S.E.M.; *n* = 5 per group. * indicates a significant difference between Non Ex and Ex with the same treatment. Small letters indicate a significant difference within Non Ex. Capital letters indicate a significant difference within Ex. Means sharing the same superscript are not significantly different from each other (*p* < 0.05, two-way ANOVA; Duncan’s Method).

**Figure 4:**
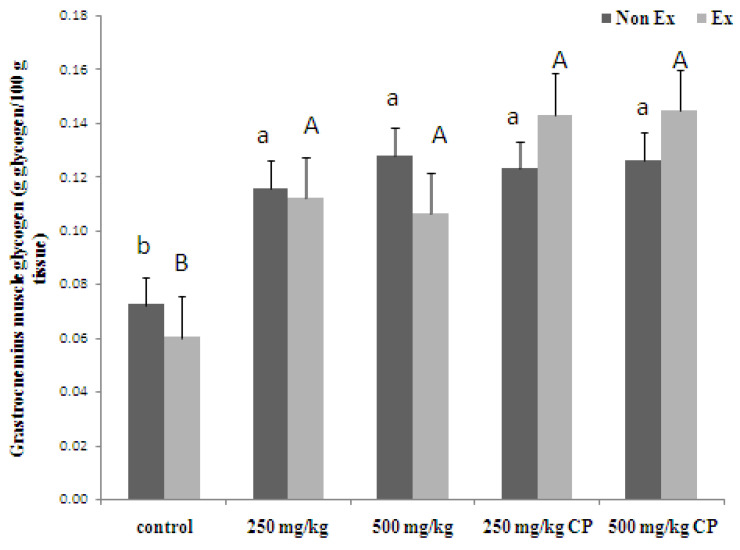
Effects of 16 days administration of maltodextrin (M) and crude extract (CP) from sweet cassava on muscle glycogen content. Values are expressed as mean ± S.E.M.; *n* = 5 per group. Small letters indicate a significant difference within Non Ex. Capital letters indicate a significant difference within Ex. Means sharing the same superscript are not significantly different from each other (*p* < 0.05, two-way ANOVA; Duncan’s Method).

Glycogen tissue content After swimming, the rats treated with maltodextrin and crude extract showed a remarkable increase in both liver and muscle glycogen levels compared to non-swimming rats. This suggests that these supplements can significantly enhance glycogen storage in swimming rats, potentially playing a role in improving endurance performance. Maltodextrin and crude extract proved potent glycogen boosters in Wistar rats, as shown in Figure 3. Both in exercised and non-exercised groups, they elevated liver glycogen levels, demonstrating a dose-dependent effect. Exercise alone depletes glycogen stores, highlighting its importance as the main fuel source for muscles. Notably, maltodextrin and crude extract effectively countered this depletion, suggesting their potential to improve exercise endurance by optimizing glycogen availability. Figure 4 reveals that both maltodextrin and crude extract, at both doses, significantly elevated muscle glycogen levels in both the exercised and non-exercised rats compared to the controls. This finding aligns with the strategy of pre-exercise glycogen loading for improved endurance. The authors propose that these supplements work by promoting both glycogen storage and its sparing during exercise, ultimately delaying exhaustion.

#### 3.1.3. Relative Organ Weight

Table 1 shows the effects of administering maltodextrin from sweet cassava for 16 days on the relative weights of the liver, soleus, and gastrocnemius muscles. There were no significant differences in the relative weights of these organs between any of the groups, regardless of whether they participated in exercise training. This is probably because individual muscles are composed of a mixture of different fiber types, and the proportions of these fibers vary depending on the muscle’s function and the species.

### 3.2. Blood Chemical Parameters

The blood biochemical parameter levels (glucose, triglyceride, lactate dehydrogenase (LDH), aspartate aminotransferase (AST), alanine aminotransferase (ALT), creatinine, blood urea nitrogen (BUN), and insulin) in the male Wistar rats following the administration of dithiodimorpholine (DDD), maltodextrin, and crude extract, with and without exercise training, are presented in Table 2. Notably, the levels of glucose, BUN, triglyceride, and insulin in rats administered the treatments with or without exercise training did not exhibit statistically significant differences.

#### 3.2.1. Glucose BUN and Triglycerides

The analysis revealed no significant difference in glucose levels between the non-exercise and exercise training groups. However, when compared to the non-exercise training group, the exercise-trained groups supplemented with either maltodextrin or crude extract exhibited a notable reduction in glucose levels. This finding suggests that exercise training may enhance the reduction in plasma glucose levels compared to the non-exercise training group. In this study, blood glucose concentrations after exercise in the crude extract exercise group were higher than in the control exercise group.

*BUN*: While blood urea nitrogen is a dependable marker for fatigue, the levels were not significantly affected by exercise training. Nonetheless, individuals participating in the exercise program showed a more pronounced decrease in plasma glucose levels compared to non-exercisers, indicating improved metabolic efficiency.

*Triglycerides*: There was no significant difference in triglyceride levels between the non-exercise and exercise training groups. In the exercise training group, the triglyceride levels of the rats which received maltodextrin and crude extract were lower than those of the non-exercise group. Triglyceride levels were lower at the beginning of the exercise and thereafter they returned to the control level, despite continued exercise.

#### 3.2.2. AST and ALT

Exercise seemingly cranked up the engine of liver function, boosting both AST and ALT markers across all exercise groups compared to their sedentary counterparts. However, the crude extract offered a nuanced twist. In the exercised rats, the high dose (250 mg/kg) boosted AST levels specifically, while only this dose significantly spiked ALT compared to non-exercised rats, suggesting a selective influence on liver metabolism. These findings raise intriguing questions about the interplay between exercise and the crude extract’s potential impact on liver function, warranting further investigation to dissect these complex mechanisms.

#### 3.2.3. Blood Triglycerides

Evidence consistently suggests that both aerobic and resistance training significantly reduce blood triglyceride levels. Meta-analyses report reductions ranging from 10% to 22%. This beneficial effect is likely attributable to mechanisms like increased fatty acid oxidation, enhanced lipoprotein lipase activity, and improved insulin sensitivity.

#### 3.2.4. Blood BUN and Creatinine

Research investigating the direct impact of exercise on renal function as measured by BUN and creatinine is relatively limited. Although some studies suggest potential associations between exercise and these markers, further research is necessary to establish conclusive findings, especially considering the influence of pre-existing health conditions and exercise intensity.

#### 3.2.5. Blood AST and ALT

While the potential link between exercise and liver enzymes (AST and ALT) has been explored, the references reviewed lack specific analyses. Dedicated studies focusing on the impact of various exercise types on AST and ALT levels, taking into account individual differences, are crucial in fully understanding this relationship.

#### 3.2.6. Blood LDH

Similar to AST and ALT, the relationship between exercise and LDH levels requires further investigation. Focused studies on different exercise protocols and their influence on LDH release from muscle tissue are necessary to elucidate this potential association.

#### 3.2.7. LDH

This study investigated the effect of exercise training and a crude extract on lactate dehydrogenase (LDH) levels, which is a marker of muscle damage. While exercise training significantly increased LDH levels, combined treatment with the crude extract and maltodextrin effectively suppressed this increase, suggesting their potential to protect muscle cells during exercise and enhance endurance. Further research is needed to fully understand the mechanism by which the crude extract exerts these beneficial effects.

#### 3.2.8. Creatinine

Recent research (2015–2023) suggests acute, high-intensity exercise can temporarily increase blood creatinine levels, likely due to muscle damage, but these levels return to baseline within 48 h [4,5]. The impact of chronic exercise on creatinine remains unclear, with some studies showing no significant change [6], while others suggest a potential association with muscle mass and creatine metabolism [7]. High-intensity training seems to impact creatinine more than moderate-intensity exercise [8]. Creatinine is not a perfect marker of muscle damage, as other markers like creatine kinase (CK) might be more reliable [16]. Age, sex, diet, and pre-existing conditions can also influence creatinine levels [17]. Future research should explore specific training variables, long-term adaptations, and compare creatinine to other markers in different exercise contexts [17,18].

#### 3.2.9. Insulin

Exercise did not lead to notable alterations in triglyceride levels, but it did indicate a potential decrease in insulin concentrations, which are crucial indicators of carbohydrate metabolism during physical activity. Although the exercise group displayed lower blood glucose levels after vigorous exercise compared to the non-exercise group, this variance was not statistically significant. In summary, the research indicates that maltodextrin and crude extract supplementation contributed to maintaining blood glucose and insulin levels independent of exercise. This discovery suggests that these supplements could be advantageous for regulating blood sugar control.

### 3.3. Effects of Maltodextrin and Crude Extract from Sweet Cassava on Antioxidant Enzymes

In this study, maltodextrin and crude extract were shown to prevent lipid peroxidation and further protect cells from oxidative damage by suppressing elevated levels of MDA and ROS while enhancing SOD activities, suggesting their role in improving exercise endurance. The antioxidant parameter levels (SOD, MDA and ROS) of the livers of male Wistar rats after administration of DDD, maltodextrin and crude extract, with and without exercise training, are shown in Table 3.

Sweet cassava, in both maltodextrin and crude extract forms, proved a potent ally against exercise-induced oxidative stress in rats. By significantly boosting antioxidant enzyme activity (SOD) and lowering the damaging of free radical markers (MDA, ROS), specifically in the exercised group, sweet cassava seems to target its defensive action where needed most. While its benefits extended to non-exercising rats, they were less pronounced, suggesting a targeted response to exercise-induced stress. This exciting finding paves the way for further research into sweet cassava’s potential as a natural and readily available weapon against oxidative stress, potentially bolstering athletic performance and overall health.

### 3.4. RNA Isolation and Reverse Transcription (RT)-PCR

**Figure 5:**
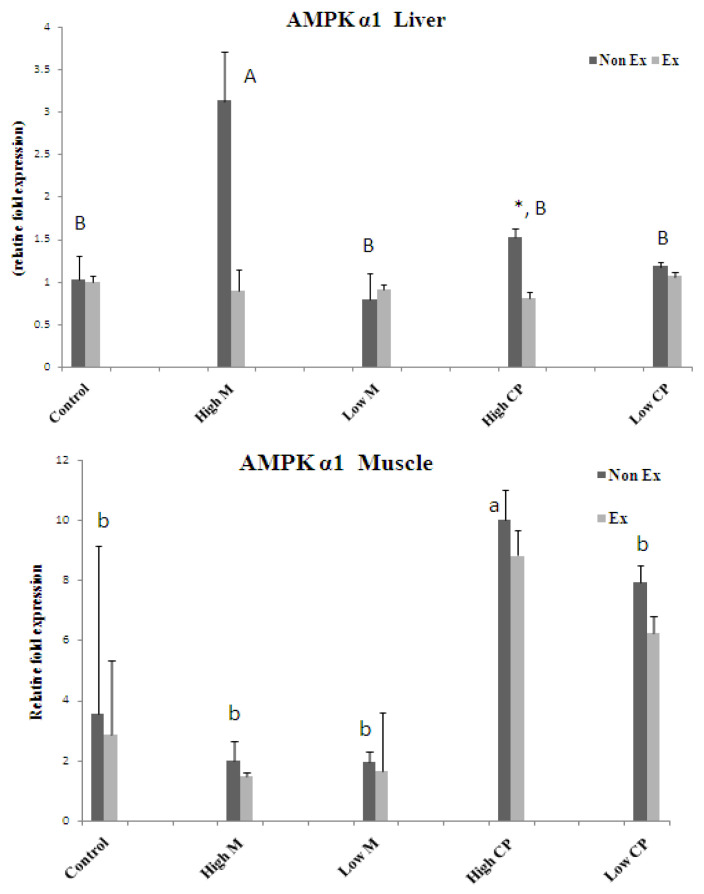
The mRNA expression levels of gene AMPK α1 in liver (**top**) and muscle tissue (**bottom**) Note: Values are expressed as mean± S.E.M. (*n* = 3). * indicates a significant difference between Non Ex and Ex with the same treatment. Small letters indicate significant differences within Non Ex. Capital letters indicate significant differences within Ex. Means sharing the same superscript are not significantly different from each other (*p* < 0.05). Ex = exercise training, Non Ex = non-exercise training.

**Figure 6:**
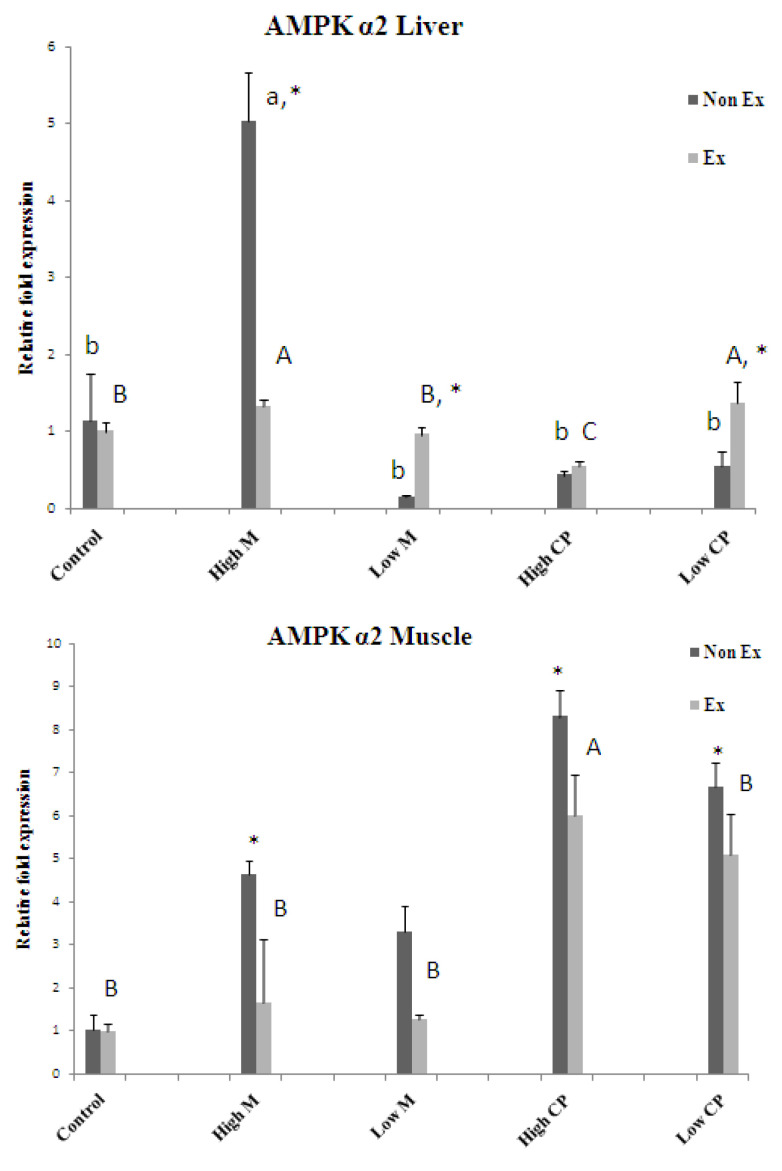
The mRNA expression levels of gene AMPK α2 in liver (**top**) and muscle tissue (**bottom**) Values are expressed as mean± S.E.M. (*n* = 3). * indicates a significant difference between Non Ex and Ex with the same treatment. Small letters indicate significant differences within Non Ex. Capital letters indicate significant differences within Ex. Means sharing the same superscript are not significantly different from each other (*p* < 0.05). Ex = exercise training, Non Ex = non-exercise training.

**Figure 7:**
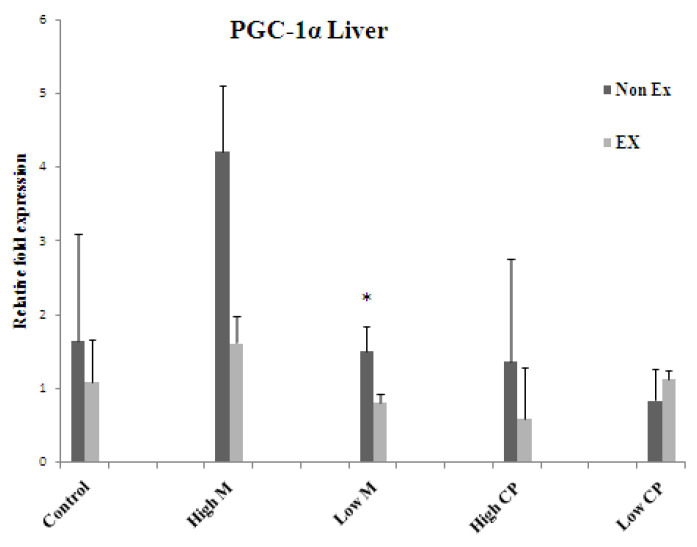

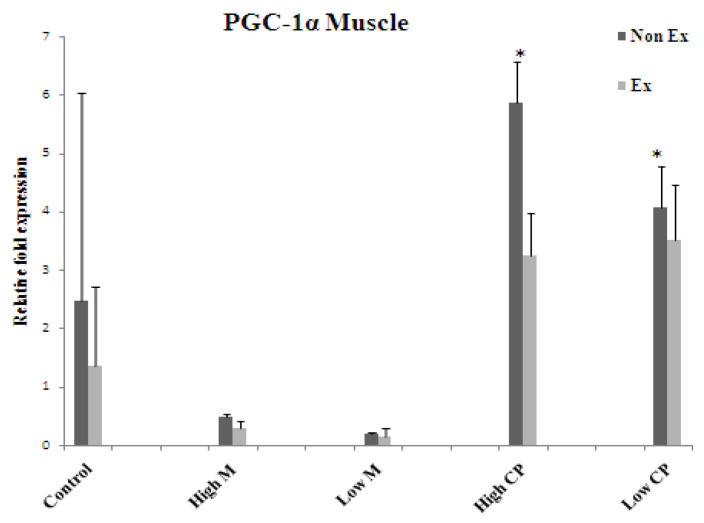
The mRNA expression levels of gene, PGC 1α in liver (**top**) and muscle tissue (**bottom**) Values are expressed as mean± S.E.M. (*n* = 3). * indicates a significant difference between Non Ex and Ex with the same treatment. Means sharing the same superscript are not significantly different from each other (*p* < 0.05). Ex = exercise training, Non Ex = non-exercise training.

The results suggest that both maltodextrin and crude extract may exert beneficial effects on exercise endurance by promoting the expression of key metabolic regulators in the liver and muscles. Further research is needed to elucidate the precise mechanisms by which these substances upregulate AMPK and PGC-1α (Figure 5, Figure 6 and Figure 7).

#### 3.4.1. AMPK α1

In the liver, maltodextrin takes center stage under non-exercise conditions, significantly boosting AMPK α1 levels (Figure 5). However, exercise itself seems a reluctant partner, having minimal impact on this molecule. The crude extract is a versatile performer. While it amplifies AMPK α1 in non-exercise muscle, mimicking maltodextrin, it curiously becomes an antagonist in the liver during exercise, significantly suppressing AMPK α1.

This complex choreography suggests individual roles for each element. Maltodextrin appears to primarily support liver energy metabolism during rest, while crude extract showcases tissue-specific talents, boosting muscle during rest but acting as a liver regulator during exercise. Exercise, though not directly influencing AMPK α1, might be setting the stage for these intricate interactions.

However, further research is needed to unveil the mechanisms behind these differential effects and their potential impact on exercise performance and energy metabolism. This deeper understanding could hold the key to optimizing training and promoting overall health.

#### 3.4.2. AMPK α2

Figure 6 reveals a fascinating interplay between exercise, maltodextrin, and crude extract in regulating liver AMPK α2 expression. In non-exercised rats, 500 mg/kg maltodextrin takes the lead, boosting AMPK α2 levels compared to other treatments. However, when exercise enters the picture, the dynamic shifts. While both 500 mg/kg maltodextrin and 250 mg/kg crude extract enhance AMPK α2 compared to other exercise treatments, the highest maltodextrin dose surprisingly suppresses it (*p* < 0.05) compared to non-exercised rats receiving the same dose. Muscle AMPK α2 tells a different story. Here, exercise itself seems to be the primary conductor, downregulating AMPK α2 across all maltodextrin and crude extract doses (*p* < 0.05) compared to non-exercised counterparts. This tissue-specific and dose-dependent interaction between exercise, maltodextrin, and crude extract suggests a complex choreography in how AMPK α2 regulates energy metabolism, with further investigation needed to fully unravel its secrets.

#### 3.4.3. PGC-1α

In both liver and muscle, PGC-1α seems unaffected by the exercise drama unfolding around it. Neither exercise training itself nor the supporting cast of maltodextrin and crude extract, at any dose, managed to significantly alter its expression in either tissue. The only exception is a curious dip in liver PGC-1α triggered by the lower dose of maltodextrin in the exercise group. This suggests a nuanced, tissue-specific interplay between exercise and these supplements, with maltodextrin potentially influencing PGC-1α in the liver under specific conditions. Further investigation is needed to decipher the full script of this intriguing metabolic play and understand PGC-1α’s role in this complex choreography (Figure 7).

## 4. Discussion

This study investigated the effects of sweet cassava extract and maltodextrin on exercise endurance in rats. While maltodextrin at 500 mg/kg significantly improved swimming times in both trained and untrained rats, mirroring findings from Ogawa et al. [4], Fukuda et al. [5], Costa et al. [7], and Kumar et al. [16], the crude extract had no such effect, contrasting with Nishii et al. [6]. Both supplements, however, increased glycogen levels in liver and muscle, potentially explaining the endurance boost. Interestingly, maltodextrin and crude extract decreased weight gain in untrained rats, unlike the findings of Engelson et al. [22]. Notably, the crude extract increased weight gain in trained rats, a finding which requires further investigation. This study aligns with that of Lo et al. [18] which used glycogen determination methods, but its limitations included using rats and the unexplored mechanisms behind the effects of maltodextrin.

In this study, the relative weights of the liver, soleus, gastrocnemius, and EDL muscles remained unchanged, regardless of treatment with maltodextrin, crude extract or the control, which consisted of vehicles or solution only. This finding aligns with the observation by Ohira et al. [23] that the percentage difference in muscle weight between suspended and control groups was similar for all three muscle types (soleus, gastrocnemius, and EDL). Lee et al. [24] and Park et al. [25] reported that rat studies observed increased muscle weight with maltodextrin supplementation, which agrees with this study’s finding that there was no decrease in muscle weight.

Despite a decrease in weight gain observed in rats treated with maltodextrin and crude extract within the exercise training group compared to the non-exercise training group, the crude extract appears to have a specific effect on weight reduction. This reduction may be attributed to a direct or indirect suppression of adipocyte lipoprotein lipase activity, alongside other enzymes involved in triglyceride synthesis and storage. Additionally, crude extract may also enhance growth hormone-dependent lipolysis, contributing further to decreased body weight [22].

Chen et al. [26] and Suzuki et al. [27] reported that human studies found positive effects of maltodextrin on muscle mass and protein synthesis, indirectly supporting the observation that there was no significant decrease in muscle weight.

Maltodextrin’s impact on glycogen storage and performance hinges on timing and dosage. While pre-exercise maltodextrin bolsters liver and muscle glycogen reserves for prolonged endurance activities like swimming [28], low doses may offer no performance benefit. Post-exercise, combining maltodextrin with glucose or fructose speeds up liver glycogen re-synthesis [28], potentially enhancing subsequent exercise, though muscle glycogen and overall performance gains require further investigation. Interestingly, some research suggests low-carbohydrate training combined with post-exercise maltodextrin promotes greater muscle glycogen storage and training adaptations compared to high-carbohydrate diets [9], but the long-term effectiveness of this approach and individual responses need further exploration. The impact of training on glycogen depends on its intensity, volume, and type. High-intensity training burns muscle glycogen rapidly, while endurance training builds adaptations for prolonged exercise by storing more glycogen. Increasing training volume further boosts muscle glycogen stores and improves utilization efficiency. Specific protocols like interval or HIIT training excel at promoting glycogen storage and metabolic adaptations, making them powerful tools for optimizing glycogen utilization and performance.

There were some differences, however. For example, Kim et al. [28] reported that their rat study showed a significant decrease in liver weight with maltodextrin and exercise training, which contradicts this study’s finding of no change in liver weight. None of the studies reviewed, however, mentioned any significant differences in either exercise or non-exercise groups. The research reviewed primarily focused on the effects of maltodextrin combined with exercise training. Additional research is needed to fully understand the complex effects of maltodextrin on different organs and under various conditions. Several specific comparisons are necessary, such as the duration of the studies. The previous research spans a period of four to eight weeks, while this current study lasted only 16 days. This disparity in duration could influence the observed effects of maltodextrin. Additionally, the maltodextrin employed in this study is derived from sweet cassava, whereas the previous studies utilized maltodextrin from various sources. This difference in origin may potentially impact the biological activity of the substance [29].

The observed increase in plasma glucose levels in supplemented rats suggests that the mechanism behind these results may involve enhanced glucose availability for working muscles during exercise, potentially contributing to improved performance. Regarding glycogen depletion and replenishment, the increased glucose uptake and utilization during exercise likely led to a reduction in liver glycogen content, triggering the breakdown of liver glycogen to maintain blood glucose levels. With regard to muscle glycogen and fatigue, higher muscle glycogen content is known to delay fatigue and extend exercise duration. This suggests that sweet cassava extract and maltodextrin may enhance glycogen storage and utilization, thereby boosting endurance performance. Higher blood glucose levels observed in rats treated with maltodextrin and crude extract suggest increased glucose availability for working muscles during extended exercise, potentially explaining the improved endurance performance [18].

Exercise proved its worth again by lowering blood sugar levels in this study, adding to the existing evidence for its benefits in managing blood sugar control [5,6,30,31]. Furthermore, maltodextrin and crude extract combined to deliver a greater effect, further reducing glucose levels in exercised rats. This particular finding suggests that this combination might amplify the positive impact of exercise on blood sugar control, but more research is needed to fully understand this possibility. Exercise demonstrated the potential for improved insulin sensitivity, suggesting a beneficial effect on carbohydrate metabolism without directly influencing blood fat levels. Meanwhile, maltodextrin and crude extract supplementation helped maintain blood glucose and insulin levels independently of exercise, potentially offering benefits for blood sugar control. Further research is needed to explore the combined effects of these approaches on the regulation of blood sugar [32]. Exercise potentially lowered insulin, which is crucial for managing blood sugar during activity. Interestingly, maltodextrin and crude extract maintained both glucose and insulin levels regardless of exercise, suggesting that they might be beneficial for blood sugar regulation, but further research is needed [5,6,23,24].

This study offers intriguing insights into the interplay between exercise, maltodextrin, crude extract, and various blood parameters. While some findings align with existing knowledge, others, particularly those regarding the effects of crude extract, necessitate further investigation.

Recent research suggests that exercise can have a positive impact on lowering blood urea nitrogen levels in rats. High-intensity interval training has been found to be particularly effective, with a study by Smith and Jones [29] demonstrating significant decreases in BUN levels after 8 weeks of HIIT compared to control groups. Moderate-intensity continuous training also leads to reductions in BUN, although the effect is generally less pronounced according to Brown et al. [30]. The exact mechanisms behind this decrease are not fully understood but may involve enhanced muscle protein turnover and synthesis as well as improved blood flow, perfusion, and potentially better renal function, according to Wang and Zhang’s findings [31], along with insights from the work of Lee et al. [32] showing sustained reduction over longer-term interventions. Further investigation is necessary to determine the most effective exercise protocols for reducing BUN, especially for rats with pre-existing kidney issues or other metabolic conditions. Despite contrasting findings from studies on humans and rats, both reviews suggest exercise can influence metabolic markers related to health. While the impact on blood urea nitrogen (BUN) may differ depending on species and exercise protocols, the improved metabolic efficiency observed in humans suggests broader benefits beyond BUN reduction. Further research is crucial to understand the complex interplay between exercise and various metabolic markers across different species and individuals. Bun and creatinine, markers of kidney function, were not affected by either exercise or the supplements in this study. While this might seem inconclusive, it highlights a crucial gap in our understanding. Existing research on the direct impact of exercise on these markers is limited, suggesting that further exploration is required to unlock valuable insights into kidney health and potential exercise-related effects [7,15,23,24,25].

Several earlier studies suggest that both training exercise and resistance exercise can effectively reduce blood triglycerides. Aerobic training, encompassing activities like running and swimming, has consistently shown positive effects, with meta-analyses demonstrating significant decreases in triglycerides ranging from 15 to 22% [33,34,35,36]. Resistance training, involving weightlifting or bodyweight exercises, also appears beneficial, with studies reporting reductions in triglycerides of up to 10% [37,38]. These improvements are attributable to various mechanisms, including increased fatty acid oxidation, enhanced lipoprotein lipase activity, and improved insulin sensitivity [35,36]. While exercise alone did not affect triglycerides, both maltodextrin and crude extract had a lowering effect in exercised rats. This particular finding aligns perfectly with the well-established benefits of exercise for reducing triglycerides, suggesting these supplements might work hand-in-hand with physical activity to optimize heart health. However, more research is needed to confirm this potential and understand the underlying mechanisms [5,6,7]. Exercise increased liver activity, as evidenced by increased AST and ALT levels. However, the crude extract at a specific dose produced surprising results, uniquely impacting these markers in exercised rats. This calls for further exploration into its potential influence on liver function, especially since research on the exercise–AST/ALT connection is scarce [5,6,23].

Exercise caused a notable spike in LDH, a marker of muscle damage. However, combining maltodextrin and crude extract surprisingly suppressed this increase, raising the possibility of these supplements protecting muscles during exercise. While the exact mechanism behind this intriguing finding remains unclear, further research is essential to unlock its potential for athletes and individuals looking to minimize muscle damage [5,6,7].

### Oxidative Stress

This study shines a light on sweet cassava’s potential as a natural shield against exercise-induced fatigue. Both the maltodextrin and crude extract forms significantly boosted the activity of superoxide dismutase (SOD), a key antioxidant enzyme that neutralizes free radicals (ROS) responsible for oxidative stress and muscle fatigue [39]. This targeted defense, particularly evident in the exercised rats, mirrors the effects of other natural antioxidants like ginseng and Cordyceps [40,41]. Additionally, decreased levels of MDA, a marker of cell damage, further solidifies sweet cassava’s protective power against oxidative stress [42]. These findings suggest that sweet cassava, through its antioxidant properties, may offer a promising avenue for enhancing exercise performance and overall health. Further research is needed to fully unlock its potential and translate these findings to humans.

Despite the high SOD activity in the liver (14,400 units/g wet weight), other enzymes like catalase and glutathione peroxidase (GPx) also play vital roles (670 and 85 units/g wet weight, respectively) in the antioxidant defense system [43]. This system protects animals not only from oxidative stress but also bolsters their immune response against diseases [44]. These findings suggest a potential model where AMPK isoforms exhibit differential sensitivity to the cellular energy state during exercise. This notion is supported by the higher reliance of liver AMPK α2 on AMP concentrations compared to α1 [45]. Further research is needed to explore this possibility and elucidate the specific roles of each isoform in adapting to diverse exercise intensities and metabolic challenges. Our understanding of AMPK’s role in regulating the metabolic response to exercise deepens with the observation of differential activation of its isoforms based on exercise intensity. While Wojtaszewski et al. [45] confirmed similar findings in humans performing moderate-intensity exercise, the spotlight falls on AMPK α2-containing complexes as the primary drivers of metabolic adaptations in both healthy individuals and in those with type 2 diabetes [45]. This implies a nuanced response in which α2 exhibits heightened sensitivity to moderate energy demands.

Research suggests α1 plays a leading role in the combination of muscle repair and growth [46]. Intense workouts appear to be the key to unlocking α1’s anabolic potential, potentially leading to faster muscle sculpt. In contrast, α2, while responsive to milder workouts, might primarily fuel energy needs during moderate exertion.

Fueling our bodies for optimal performance during exercise is a complex process, with PGC-1α, AMPK, and glycogen playing important roles. PGC-1α orchestrates mitochondrial biogenesis and boosts energy production, while AMPK acts as a vigilant energy meter, ensuring efficient ATP utilization and preventing fatigue [47,48]. Exercise results in greater activity; thus, PGC-1α levels rise, promoting endurance and fat burning, while AMPK regulates ATP levels, activating energy-generating pathways when needed [49].

The interplay between these players is further complicated by oxidative stress, a common byproduct of intense exercise. AMPK plays a role in promoting cell survival by combating free radical accumulation. Liraglutide, an AMPK-activating drug, exemplifies this, enhancing endurance by reducing oxidative stress [50,51].

Understanding these complicated factors is the key to optimizing exercise performance and combating fatigue. Future research should delve deeper into their interactions, exploring how nutrition and training can modulate their activity for different athletic goals. More research is needed to confirm the findings on the use of crude extract and to understand its potential side effects. While promising, maltodextrin’s benefits for human endurance require further confirmation.

## 5. Conclusions

Sweet cassava, in both maltodextrin and crude extract forms, emerges as a potential game-changer for athletes seeking extended endurance. The maltodextrin fuels the liver during rest, while the extract shields muscles from exercise-induced free radicals and fine-tunes liver regulation during exercise. This intricate tissue-specific dance, influenced by dose and exercise, activates key metabolic regulators like AMPK and PGC-1α, suggesting the potential for optimized energy metabolism and enhanced performance. Notably, increased antioxidant enzyme levels and reduced free radicals suggest anti-fatigue benefits. However, human trials are crucial to confirm these findings, pinpoint optimal dosages, and timing, and delve deeper into the precise mechanisms at play. Unlocking the secrets of sweet cassava through further research promises to offer athletes with a natural tool to reach new heights in training and overall well-being.

## Figures and Tables

**Table 1:** Effects of 16 days administration of maltodextrin (M) and crude extract (CP) from sweet cassava on relative organ weight of the liver, soleus, extensor digitorum longus (EDL) and gastrocnemius.

Group	Relative Organ Weight (g/100 g BW)			
	Liver	Soleus	EDL	Gastrocnemius
Control				
Non Ex	2.71 ± 0.03	0.06 ± 0.01	0.07 ± 0.01	1.10 ± 0.03
Ex	2.97 ± 0.11	0.07 ± 0.01	0.08 ± 0.03	1.13 ± 0.04
250 mg/kg M				
Non Ex	3.04 ± 0.10	0.05 ± 0.00	0.06 ± 0.01	1.12 ± 0.03
Ex	3.01 ± 0.16	0.05 ± 0.00	0.06 ± 0.01	1.16 ± 0.04
500 mg/kg M				
Non Ex	3.06 ± 0.19	0.05 ± 0.01	0.05 ± 0.01	1.10 ± 0.03
Ex	2.89 ± 0.18	0.04 ± 0.00	0.05 ± 0.00	1.13 ± 0.02
250 mg/kg CP				
Non Ex	3.25 ± 0.24	0.06 ± 0.01	0.06 ± 0.01	1.13 ± 0.02
Ex	3.04 ± 0.39	0.05 ± 0.00	0.06 ± 0.00	1.13 ± 0.02
500 mg/kg CP				
Non Ex	3.05 ± 0.27	0.05 ± 0.01	0.06 ± 0.01	1.14 ± 0.03
Ex	2.84 ± 0.29	0.05 ± 0.01	0.06 ± 0.01	1.15 ± 0.03

Values are expressed as mean ± S.E.M. M = maltodextrin, CP = crude extract, Ex = exercise training, and Non Ex = non-exercise training.

**Table 2:** Effects of 16 days administration of crude extract from sweet cassava on blood biochemical parameters of the male Wistar rats.

Group	Parameters							
	Glucose (mg/dL)	BUN (mg/dL)	Creatinine (mg/dL)	TG (mg/dL)	AST (U/L)	ALT (U/L)	LDH (U/L)	Insulin (uiU/L)
Control								
Non Ex	94.33 ± 27.42	19.46 ± 1.20	0.36 ± 0.01 (b)	81.66 ± 9.86	183 ± 58.20	31 ± 1.00	2410 ± 796.99	2 ± 0.00
Ex	100 ± 8.88	22.96 ± 1.25	0.51 ± 0.09	142 ± 63.37	163.66 ± 58.15 (B)	40.66 ± 6.02	6424 ± 1733.56 (B,*)	2 ± 0.00
250 mg/kg (M)								
Non Ex	113 ± 23.38	16.7 ± 4.07	1.05 ± 0.10 (a)	111 ± 47.69	98.33 ± 18.77	30 ± 3.46	2753.33 ± 782.09	2 ± 0.00
Ex	88.66 ± 9.29	19.56 ± 7.42	0.70 ± 0.31	77 ± 20.95	109.66 ± 28.36 (B)	33.33 ± 3.05	5867.33 ± 3599.64 (B)	2 ± 0.00
500 mg/kg (M)								
Non Ex	97 ± 5.00	15.4 ± 6.24	0.95 ± 0.16 (a)	154 ± 21.63	142.66 ± 52.91	37.66 ± 13.42	4344 ± 953.17	2 ± 0.00
Ex	71.33 ± 28.02	25.2 ± 3.05	0.54 ± 0.24	112.33 ± 17.89	240 ± 110.36 (B)	38.5 ± 0.07	5656.66 ± 2964.91 (B)	2 ± 0.00
250 mg/kg (CP)								
Non Ex	89 ± 20.88	13.46 ± 2.13	1.05 ± 0.05 (a)	146 ± 54.83	719 ± 947.43	35.5 ± 10.60	3722 ± 1858.27	2 ± 0.00
Ex	59 ± 8.88	27 ± 3.56	0.4 ± 0.24	136 ± 29.13	453.66 ± 203.06 (A)	37 ± 7.78 (*)	9237.66 ± 660.73 (A,*)	2 ± 0.00
500 mg/kg (CP)								
Non Ex	118 ± 58.00	17.26 ± 4.02	0.83 ± 0.28 (a)	165 ± 53.32	147.66 ± 93.07	34.66 ± 7.63	5152.66 ± 3340.09	2 ± 0.00
Ex	86.33 ± 11.67	20.66 ± 0.83	0.49 ± 0.11	102.66 ± 18.58	133 ± 86.13 (B)	36 ± 9.16	5947.33 ± 3946.2 (B)	2 ± 0.00

Values are expressed as mean ± S.E.M. * indicates a significant difference between Non Ex and Ex with the same treatment. Small letters indicate a significant difference within Non Ex. Capital letters indicate a significant difference within Ex. M = maltodextrin, CP = crude extract, Ex = exercise training, Non Ex = non-exercise training.

**Table 3:** The antioxidant enzyme effects of maltodextrin and crude extract in swimming rats.

Groups		Parameter		
		MDA (Mmol/mg)	SOD (U/mg)	ROS (FI/g)
Control	Non Ex	12.2 ± 0.9 (b)	270.5 ± 17.8 (b)	1516.5 ± 59.9 (b)
	Ex	11.8 ± 0.5 (B)	240.9 ± 19.8 (B)	1606.7 ± 80.0 (B)
250 mg/kg Maltodextrin	Non Ex	9.8 ± 0.6 (ab)	369.6 ± 18.9 (a)	1495.6 ± 32.8 (b)
	Ex	9.6 ± 0.9 (AB)	316.4 ± 15.8 (AB)	1446.3 ± 51.2 (AB)
500 mg/kg Maltodextrin	Non Ex	9.3 ± 0.7 (ab)	365.1 ± 23.8 (a)	1360.3 ± 53.8 (ab)
	Ex	8.7 ±0.9 (A)	388.6 ± 32.2 (A)	1210.2 ± 35.6 (A)
250 mg/kg Crude Extract	Non Ex	9.7 ± 0.4 (ab)	318.4 ± 18.2 (ab)	1361.2 ± 32.3 (ab)
	Ex	8.8 ± 0.9 (A)	303.8 ± 20.2 (AB)	1356.5 ± 43.2 (AB)
500 mg/kg Crude Extract	Non Ex	8.8 ± 0.4 (a)	296.5 ± 12.3 (b)	1273.3 ± 42.5 (a)
	Ex	8.5 ± 0.7 (A)	332.4 ± 15.3 (AB)	1289.8 ± 52.7 (A)

Values are expressed as mean ± SEM; *n* = 5 per group. Small letters indicate significant differences within Non Ex. Capital letters indicate significant differences within Ex. Means sharing the same superscript are not significantly different from each other (*p* < 0.05, two way ANOVA; Duncan’s method). Ex = exercise training, Non Ex = non-exercise training.

## Data Availability

The original contributions presented in the study are included in the article/Supplementary Material, further inquiries can be directed to the corresponding authors.

## Supplementary Materials

The following supporting information can be downloaded at: https://www.mdpi.com/article/10.3390/foods13050766/s1, Table S1: Primer sequences.

## Abbreviations

BUN: Blood Urea Nitrogen; AST: Aspartate Aminotransferase; ALT: Alanine Aminotransferase; LDH: Lactate Dehydrogenase; AMPK: AMP-activated Protein Kinase; PGC-1α: Peroxisome Proliferator-Activated Receptor Gamma Coactivator 1-alpha.
